# Healthcare Professionals’ Ethical Responsibility in COVID-19 Vaccination Decision-Making

**DOI:** 10.21315/mjms2022.29.2.14

**Published:** 2022-04-21

**Authors:** Yusrita ZOLKEFLI

**Affiliations:** Pengiran Anak Puteri Rashidah Sa’adatul Bolkiah (PAPRSB) Institute of Health Sciences, Universiti Brunei Darussalam, Brunei Darussalam

**Keywords:** vaccination, healthcare, refusal, ethics, responsibility, professionals

## Abstract

Healthcare professionals’ decision about whether to receive COVID-19 vaccination is grounded in fundamental ethical bases. This paper considers some of the ethical responsibilities surrounding vaccination. While healthcare professionals have the right to refuse the vaccine, they are urged to reflect on three key responsibilities in making the decision: i) professional responsibility; ii) social responsibility and iii) personal responsibility within the ethical dimension. This paper also argues that, in promoting vaccine acceptance, healthcare organisations have a greater institutional responsibility to be transparent and keep their staff informed about the vaccine to the best of their ability. A balanced and harmonised ethical responsibility of healthcare professionals must be critically considered in making vaccination decisions.

## Introduction

The COVID-19 pandemic has put many healthcare facilities, systems and even entire societies under unprecedented stress levels. There are many different descriptions and reports of the healthcare professionals’ perception of and willingness to receive COVID-19 vaccinations (1–2). Some reports estimate that the rates of COVID-19 vaccination hesitancy among healthcare professionals may be similar to the rates in the general population (3). Potential contributors to vaccine hesitancy among healthcare professionals include safety concerns, doubts about effectiveness and perceived low risks of infection among those who do not treat patients with COVID-19 (4). In a recent report, more than 150 hospital workers at Houston Methodist Hospital were fired or resigned after refusing to follow a policy that requires employees to be vaccinated against COVID-19 (5). Despite various practical solutions to the hesitancy and refusal, such as education, the reports suggest a tension between individual choice and ethical responsibility (6). There seems to be a sense of the interdependence between individuals’ responsibility to be vaccinated, collective responsibility to realise herd immunity against infectious diseases, and institutional responsibility to enact policies that guarantee herd immunity (6). This paper will not examine the causes of hesitancy or refusal in any detail since its purpose is to focus on the ethical dimension of vaccination decisions. The paper explores the basis for the healthcare professional’s ethical responsibility to be vaccinated.

It is helpful to define the term ‘refusal’ and to distinguish it from other concepts in healthcare ethics. The key point to observe is that there is a difference between refusing treatment as an individual and refusal in the public interest. A generally established point of healthcare ethics is that a competent patient can refuse medical treatment, including lifesaving treatment (7). For example, suppose the doctor advises the patient to undergo surgery to remove a tumour. After careful consideration, the patient refuses. Such a refusal is unlikely to affect the public health interest directly. However, the refusal of treatment can significantly influence other people’s health when the refusal comes from a patient with a highly infectious condition. The coronavirus is a prime example.

## Ethics and Professional Responsibility: Part 1

Healthcare professionals have made a professional commitment to protecting patients; that is, the commitment to ‘do no harm’. This responsibility is reflected in professional codes like the Hippocratic Oath and the codes of various medical and allied health associations. This responsibility obliges healthcare workers to prevent harm. In the context of the pandemic, this means avoiding actions that could expose patients to the virus and following reasonable precautions. It can be argued that refusing to be vaccinated violates the responsibility not to harm and place others at significant risk of harm (8). A healthcare professional may be infected without showing any symptoms and transmit the virus to patients, posing a public health risk. However, the question of the probability of harm must also be critically considered.

One might argue that the risk of harm from unvaccinated professionals is uncertain. In other words, the harm is not imminent. While healthcare workers are at increased risk of virus exposure while caring for COVID-19 patients, the precise epidemiological data regarding such transmission are currently scarce (9). However, the latest study (10) shows that the harm to society caused by an unvaccinated healthcare professional is, in fact, imminent, particularly with the emerging variants such as delta. Preliminary evidence from Scotland suggests that people infected with delta variant are about twice as likely to end up in the hospital as those infected with alpha variant (11). Meanwhile, fully vaccinated healthcare employees and other frontline workers in the United States were reported to be 25 times less likely than unvaccinated persons to test positive for COVID-19 (12). Such results suggest that vaccinated individuals are highly protected from being infected and, as a result, are unlikely to spread the virus. Furthermore, studies have found that people who tested positive for COVID-19 after getting their first vaccine dose had lower virus levels in their bodies than unvaccinated people who tested positive (13). The decreased viral load hints that vaccinated people who do contract the virus would be less infectious, because they would have a much smaller amount of the virus to spread (14).

Additionally, COVID-19 superspreading events in healthcare settings have been documented globally (15–16). Hospitals are an essential setting for viral transmission (17). This makes hospitals and clinics a point of disease transmission, where healthcare professionals are at increased risk of contracting infections. The World Health Organization has recently suggested that healthcare professionals account for up to 1 in 7 cases of COVID-19 worldwide (18). Maximising COVID-19 vaccinations among healthcare professionals could help reduce superspreading events (19).

When a healthcare professional enters the healthcare community, he or she makes a specific commitment to upholding the patients’ interest and improving the patients’ health. This includes recognising that the virus can make anyone seriously ill. However, the risk is higher for people who cannot protect themselves, such as immunocompromised patients. COVID-19 mortality shows a strong relationship between age and pre-existing medical conditions (20). Furthermore, healthcare professionals regularly work with and treat vulnerable populations, including older and immunocompromised individuals; this heightens the importance of infection prevention, including via uptake of safe and effective vaccines to prevent transmission to patients (21). In spite of all this, a healthcare professional could be ethically justified in breaking his or her promise to patients if keeping the promise were impossible or highly impractical. For example, if a doctor could not be vaccinated for medical reasons, it would be acceptable for this doctor to remain unvaccinated.

## Ethics and Professional Responsibility: Part 2

Healthcare professionals also have an ethical responsibility to the profession: they are responsible for keeping working conditions as safe as possible, particularly in times of crisis. This notion is important for two reasons: promoting patient trust and protecting the entire healthcare system. We know that healthcare professionals are required to practice the profession with honesty, integrity, and accountability to maintain the patients’ confidence and society at large. Furthermore, as a profession that society has invested with trust, power, and authority over public health and interest, healthcare professionals have a unique role and a great ethical responsibility to maintain high professional standards. Healthcare professionals should strive to safeguard and preserve professional integrity by not engaging in activities inconsistent with the ethical norms and obligations of the profession. They are also accountable for their professional behaviour, activities, and actions. They must not do anything to jeopardise the functioning of the health sector. The success of the pandemic response depends on the broad support of all people, including healthcare professionals, in fulfilling the duties for the benefit of society at large. This means that all healthcare professionals must be ready to endorse vaccination and become role models to encourage people to get vaccinated. This requires healthcare professionals to communicate responsibly, particularly in the public arena. However, they cannot ‘walk the talk’ if they choose to refuse the vaccine.

Healthcare professionals also have an ethical responsibility to their colleagues: they are more likely to spread the virus to colleagues if they do not get vaccinated. Such an act can undermine the value of respect for colleagues (looking out for and helping each other); it can be argued that it is ethically wrong to risk transmitting the virus to colleagues when a vaccine is available. However, it can also lead to more significant damage, creating a sense of disharmony and breaking down trust between the healthcare professionals themselves.

Healthcare professionals also have a right to be protected from occupational infection (22). Professional virtues exist to ensure a good workplace relationship. Such virtue places an ethical responsibility on healthcare professionals to be immune, especially when they are on the front line and urgently needed. The pandemic has exacerbated shortages in healthcare staffing and resources (23). One of the reasons healthcare professionals are highly prioritised to receive COVID-19 vaccines is to maintain healthcare staffing. In order for that to happen, healthcare professionals should not do anything that may place themselves at risk. This responsibility is clearly stated in most codes of professional conduct, whereby healthcare professionals are responsible for promoting their own personal health, safety and well-being. Healthcare professionals’ well-being matters for two broad reasons. First, healthcare professionals are human beings whose well-being counts as much as everyone else’s; second, healthcare professionals are needed to provide patient care (24). Particularly in a pandemic, patients would suffer harm if healthcare professionals were physically or mentally unable to do their jobs. Healthcare professionals are one of the scarcest resources in this pandemic and are needed to save more lives through their work at the bedside. Therefore, it is crucial to protect the lives of healthcare professionals and maintain the safest working conditions possible during this time of crisis.

## Ethics and Social Responsibility

The second type of ethical responsibility of healthcare professionals is towards society at large. Healthcare professionals have a social responsibility to limit the spread of diseases. This responsibility can save healthcare facilities and systems from being overburdened. This is particularly true now, when the pandemic has exacerbated healthcare staffing and resources (23). A vaccinated healthcare professional is more likely to act as a barrier against the spread of infection, especially in primary healthcare delivery during outbreaks. In deliberating this responsibility, we must acknowledge the principle of utilitarianism, that is, the greatest happiness for the greatest number of people. From a utilitarian perspective, we must observe the interest of other people by recognising that a herd community cannot be established efficiently if many people in the community refuse the vaccine. Refusal for reasons such as concern that the vaccine itself may be harmful, may only benefit the individual. However, the consequences of the refusal can trickle down to many others in the community. Infectious disease outbreaks can be very disruptive to everyday life and have a substantial economic cost (7).

Another critical point to reflect on is the value of saving a life. Healthcare professionals may claim that requiring everyone to get vaccinated is ethically questionable because they cannot prevent everyone from contracting the infection. One may also argue that individual actions make no difference and cannot stop the pandemic. However, these arguments are weak for two reasons. First, the chance to save a life by preventing a small transmission of infection is ethically commendable, whereby every healthcare professional must not contribute to collective harm. Secondly, the principle of distributive justice requires fairness in contributing to public goods such as healthcare resources. It is unfair for unvaccinated healthcare professionals to act as free riders who use the public good without assuming a fair share of the cost. The free riders are happy for others to suffer the side effects of the vaccine but unwilling to do their part to protect the community. Recalling the tragedy of the commons, one can argue that if only a small number of individuals refuse the vaccine, the situation is nuanced. However, if many refuse, this can erode herd immunity and cause the entire community to become vulnerable to pandemic outbreaks. This prospect is ethically unacceptable. For the principle of justice to work well, there must be some sense of community solidarity, where each member assumes a small risk to protect the community through vaccination.

## Ethics and Personal Responsibility

We must note that there are no absolute rights and no absolute autonomy. No one can survive or flourish without the help of others. To put it simply, all people have been helped by others; the idea of rugged individualism (25) is a myth, where dogmatic views of individual autonomy are misguided and inaccurate. Everyone’s autonomy in society is limited to avoid harming oneself or others. The pandemic has made it even more evident that we rely on others to function in society. For example, scientific research has informed our knowledge of the virus. These contributions support the common good. On the other hand, individualism can lead to a disregard for the worth and value of others, where individuals become unwilling to sacrifice a degree of their freedom to protect themselves or others.

Meanwhile, healthcare professionals advocate for the best evidence-based practices. Therefore, healthcare professionals’ decisions on vaccination should be drawn from current evidence. This means that professionals are responsible for promoting health and safety by being sufficiently informed about vaccination. This responsibility means a commitment to understand, support, and adhere to the current evidence-based standards and guidelines. If healthcare professionals do not have sufficient knowledge, they must seek reliable information. They must also verify the information they find and accept the scientific evidence as openly as possible. Fear of vaccination is impossible to defend since any reasonable fear can be addressed through learning about the vaccine and reviewing scientific evidence. Evidence suggests that improving knowledge through training also improves vaccine confidence in healthcare professionals. How can a patient be expected to consult a healthcare professional with confidence when the profession cannot offer concrete confirmation about the healthcare professional’s own health? Furthermore, for a healthcare professional, to value his or her personal choice over professional responsibility would be incompatible with being a healthcare professional.

## Conclusion

It may be naïve to expect all healthcare professionals to unquestioningly accept and trust COVID-19 vaccination. However, without ignoring the principles of personal choice, we must embrace and consider accepting the vaccine a responsibility, even if the decision is difficult. At the same time, healthcare institutions should be more willing to engage with healthcare professionals’ lived experiences, take their ethical concerns seriously (19), gain the trust of healthcare professionals (26) and run robust educational campaigns among the staff to promote voluntary vaccination (27). Meanwhile, to achieve this common goal, healthcare organisations and healthcare professionals must acknowledge their duties to each other. Healthcare organisations are responsible for making the vaccination program transparent, while healthcare professionals are responsible for being informed and accepting the scientific evidence when making the vaccination decision. A recent study reported that providing information on personal benefits may reduce hesitancy to a greater extent than information on collective benefits (28).

In summary, during the current pandemic, healthcare professionals must weigh professional, social, and personal responsibilities when deciding whether to get vaccinated. Healthcare professionals are responsible for ensuring adequate and concerted efforts to promote the public good. Healthcare professionals are, first and foremost, members of the healthcare community, where they need to be able to adopt a sensible approach to promoting public health interest. It is important to remember that a healthcare professional’s vaccination decision should not be premised on the concept of personal choice and individual rights because this can later lead to an oversight of ethical responsibility. Meanwhile, there is no doubt that the healthcare organisation has the institutional responsibility to be transparent in its vaccination efforts. All in all, when making vaccination decisions, healthcare professionals must consider a balanced and harmonised ethical responsibility, as well as committed to upholding sustainable public health practices. The COVID-19 pandemic has certainly highlighted the importance of healthcare professionals always striving to do the right thing, respecting the common good, and recognising and accepting one another’s vulnerability and interdependence.
